# Practice and Perceptions on Extracorporeal Carbon Dioxide Removal in the Current Era: A Multinational Survey

**DOI:** 10.1111/crj.70203

**Published:** 2026-06-16

**Authors:** Ravindranath Tiruvoipati, Andrew Wang, Matthew E. Cove, Son Do, QuangDai Huynh, Simon Sin Wai Ching

**Affiliations:** ^1^ Department of Intensive Care Medicine Frankston Hospital Frankston Victoria Australia; ^2^ Division of Medicine, Peninsula Clinical School Monash University Frankston Victoria Australia; ^3^ ANZIC‐RC, School of Public Health and Preventive Medicine Monash University Melbourne Victoria Australia; ^4^ Department of Medicine, Division of Respiratory and Critical Care Medicine National University Hospital Singapore Singapore; ^5^ Center for Critical Medicine, Bach Mai Hospital University of Medicine and Pharmacy, Vietnam National University Hanoi Vietnam; ^6^ Department of Critical Care, Emergency Medicine and Clinical Toxicology University of Medicine and Pharmacy Ho Chi Minh City Vietnam; ^7^ Critical Care Medicine Unit, School of Clinical Medicine University of Hong Kong Hong Kong China

**Keywords:** ARDS, asthma, COPD, extracorporeal carbon dioxide removal, registry, survey

## Abstract

**Introduction:**

Extracorporeal carbon dioxide removal (ECCO_2_R) is a type of partial extracorporeal respiratory support with potential to reduce lung injury in patients with hypercapnic respiratory failure. We aimed to study the current practice and perceptions of ECCO_2_R and the need for an ECCO_2_R registry.

**Methods:**

A multinational survey was conducted among clinicians who have published clinical studies on ECCO_2_R or have used ECCO_2_R within the last 5 years in Europe, Asia, Oceania and the United States.

**Results:**

Seventy responses from 140 requests (response rate: 50%) were received from physicians across all targeted regions. ECCO_2_R is reported to be used mainly for ARDS (46%) management, followed by asthma (18%) and COPD (15%). Eighty‐five percent of the participants reported low case volumes (0–10 cases per year). Variations in the practice of ECCO_2_R were evident, including triggers for initiating ECCO_2_R, devices used, access catheter type and size, targeted blood flow rates and monitoring of anticoagulation. The most common barriers to wider use of ECCO_2_R included lack of evidence, followed by the device's costs and operational complexity. Eighty‐four percent (48/57) of the respondents thought an ECCO_2_R registry would be useful, and 98% (47/48) of the respondents reported that they would be willing to contribute to a dedicated ECCO_2_R registry.

**Conclusion:**

ECCO_2_R practices varied considerably, but most respondents agreed on the usefulness of a dedicated ECCO_2_R registry. Practice variations may be associated with suboptimal outcomes. ECCO_2_R quality indicators are needed to optimise clinical outcomes and resource utilisation.

## Graphical abstract

This multinational survey of ECCO_2_R clinicians showed that ECCO_2_R practices varied considerably, but most participants agreed on the usefulness of a dedicated ECCO_2_R registry. Practice variations may be associated with suboptimal outcomes. ECCO_2_R quality indicators are needed to optimise clinical outcomes and resource utilisation.

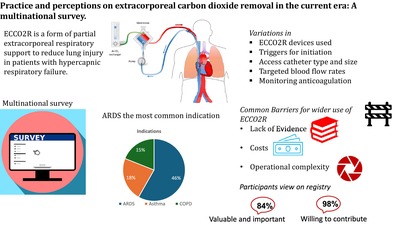

## 1 Introduction

Extracorporeal carbon dioxide removal (ECCO_2_R) is a type of extracorporeal life support (ECLS) useful in patients with hypercapnia, resulting from either Type II respiratory failure or adjustments to mechanical ventilator settings to achieve lung protective ventilation [1, 2]. While ECCO_2_R has been in clinical use for four decades, technological advances have resulted in an array of commercially available devices with different trade‐offs in terms of efficiency and complication profile [1]. Many observational studies have shown ECCO_2_R to be effective in reducing PaCO_2_ and improving pH in patients with ARDS, COPD and asthma [3, 4, 5]. However, while significant, the reported improvements in pH and PaCO_2_ appear variable, perhaps reflecting that most studies are small case series [2, 4, 6]. Recent randomised controlled trials, which included ARDS and COPD patients, have failed to demonstrate a significant benefit in improving the survival of patients or reducing the need for mechanical ventilation in those who were treated with ECCO_2_R [7, 8]. Furthermore, in the REST trial, ECCO_2_R was not associated with improvements in long‐term outcomes, including 1‐year mortality, respiratory function, post‐traumatic stress disorder, cognitive dysfunction or health‐related quality of life, and was associated with higher costs and potentially significant complications [9, 10, 11]. While well conducted, the randomised controlled studies had important limitations, including a lack of experience at participating centres, case selection and stopping recruitment before reaching the planned sample size [12, 13]. The implications of these trials on the current practice and perceptions of low flow ECCO_2_R use by clinicians are currently not known.

There are no dedicated ECCO_2_R registries similar to ELSO (extracorporeal life support organisation) or INDEX (INTERNATIONAL RESEARCH DATABASE FOR EXTRACORPOREAL SUPPORT) to help in supporting ECCO_2_R clinical research, assist regulatory agencies on quality of care or facilitate individual healthcare settings in making decisions on patient care and establishing treatment protocols. Recently, INDEX registry has included ECCO_2_R in conjunction with ECMO patients in an attempt to fill this gap but does not appear to be used as widely as ECMO registries.

We aimed to characterise the current practices and perceptions regarding ECCO_2_R among clinicians who have either published clinical studies on ECCO_2_R or who are currently using, or have used, the technology within the past 5 years. A further objective was to explore their views on the need for, and potential value of, an ECCO_2_R registry.

## 2 Materials and Methods

We conducted an online/web‐based survey to assess the practices of ECCO_2_R in centres across the world. An online survey of 20 questions was designed (refer to attached invitation request [Appendix S1] and Survey [Appendix S2]) to capture basic demographic data, followed by questions on ECCO_2_R practice and the usefulness of an ECCO_2_R registry. The survey was pretested within a focus group of 12 ECCO_2_R clinicians to refine the survey by identifying ambiguous questions or wording. The survey was then conducted using the online survey tool Qualtrics Plus via the Helix platform offered and supported by Monash University. Participants were able to skip any question they did not wish to answer and proceed to the next item.

Potential participants were identified through published clinical ECCO_2_R studies (January 2019–April 2024), direct contact with clinicians experienced in ECCO_2_R, consultation with local experts and information from ECCO_2_R device manufacturers regarding centres using ECCO_2_R. Authors reporting only in vitro ECCO_2_R studies or animal experiments were excluded from the survey. Clinicians, who had experience in extracorporeal membrane oxygenation (ECMO), but not with low flow ECCO_2_R, were also excluded from this survey, as the main aim of the survey was to obtain insights related to low flow/intermediate flow ECCO_2_R and not total extracorporeal supports such as ECMO.

Identified participants were contacted through email or telephone (where available) to request them to participate in the survey. For nonresponders, two reminders were sent about 3 weeks apart. No further reminders were sent, and the results that were available were analysed. The survey was open between 20th June 2024 and 19th September 2024.

### 2.1 Ethical Considerations

Ethics approval was obtained from the Human Research and Ethics Committee of Peninsula Health (HREC Reference number: LNR/108005/PH‐2024; SSA Reference number: LNRSSA/108005/PH‐2024).

### 2.2 Statistical Analyses

The data analyses were primarily descriptive and reported as mean, median and range as appropriate. Categorical data are reported as percentages of valid responses; hence, the denominator of the response is variable depending on the number of valid responses. For questions allowing multiple responses, the total number of responses for each item was included in the denominator.

## 3 Results

**FIGURE 1:**
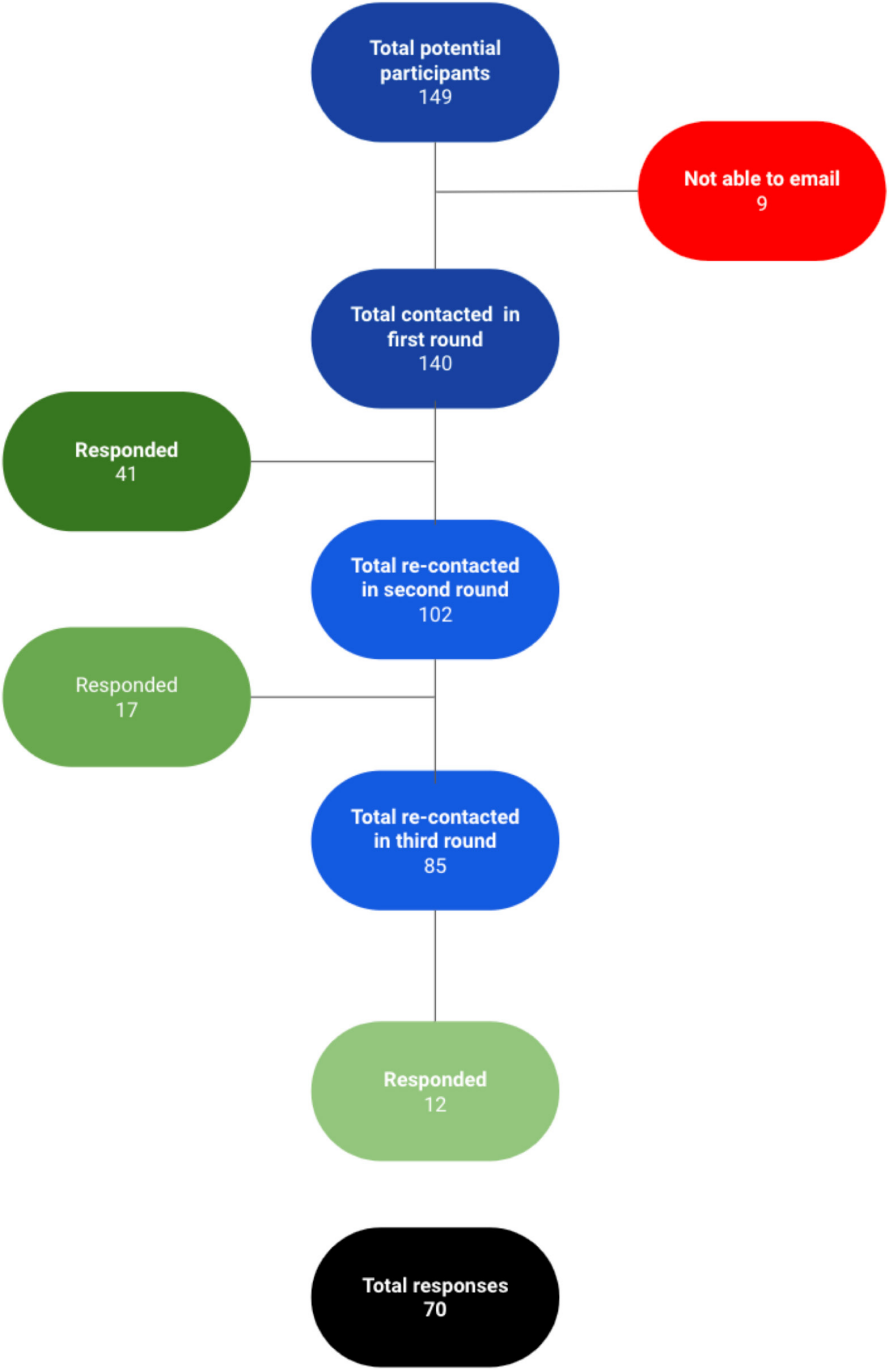
Study profile.

Of the 149 identified clinicians, 140 clinicians were contacted by email. The email available for nine clinicians was not accurate, and no other contact details were available. Of the 140 clinicians contacted, 70 responses were obtained giving a response rate of 50% (Figure 1).

### 3.1 Demographics

**FIGURE 2:**
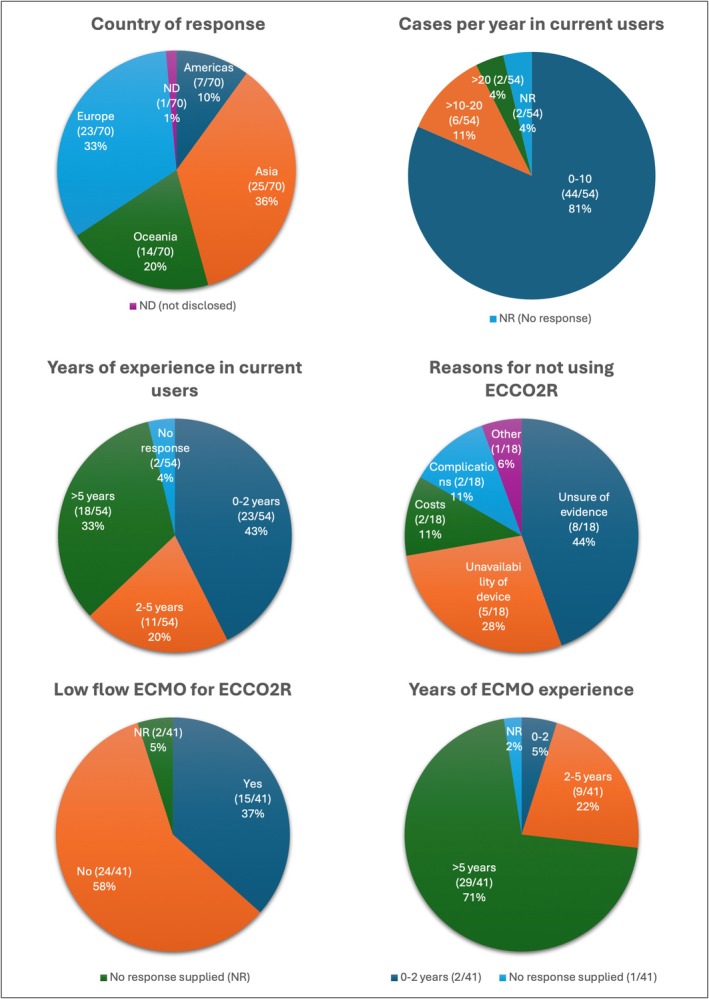
Demographic and experience of ECCO_2_R in survey participants.

**FIGURE 3:**
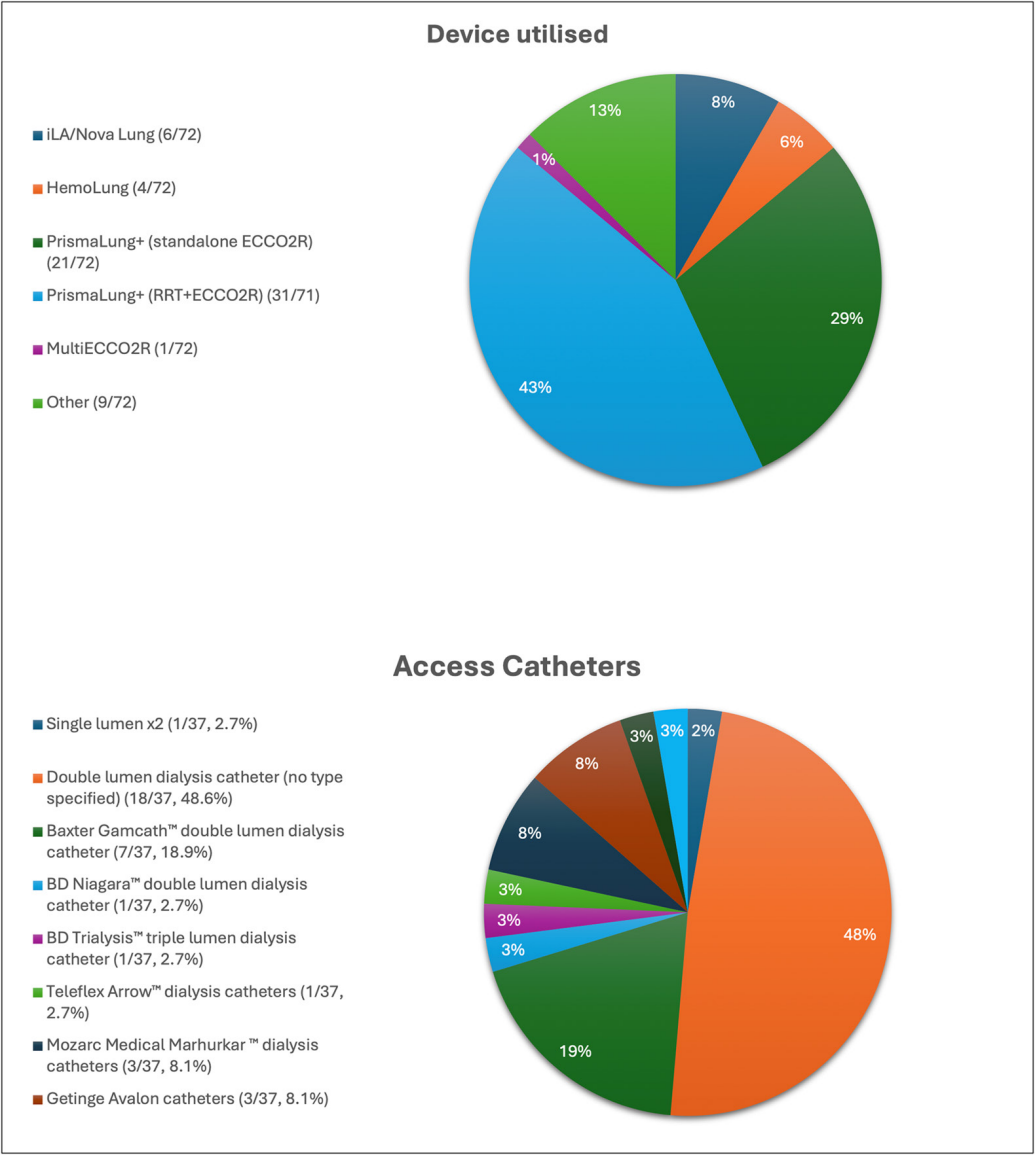
ECCO_2_R devices and access catheters currently used.

The participants were mainly from Europe, followed by Asia (Figure 2). More than 77% (54/70) of the responders were currently using ECCO_2_R. Of the 23% (16/70) who were not currently using ECCO_2_R, the main reasons included lack of evidence (44%), lack of device (28%), cost (11%) and complication fears (11%). Most current ECCO_2_R users reported experience of less than 2 years in using ECCO_2_R, in centres with a case volume of < 10 cases per year (Figure 2). Nearly 60% of the participants were using ECMO as well as ECCO_2_R in their centre. PrismaLung+ (Baxter Healthcare Pty Ltd) was the most commonly used device among participants, enabling ECCO_2_R to be combined with continuous renal replacement therapy (Figure 3). Some respondents reported access to more than one device type within their ICU; consequently, the denominator for this item exceeded the total number of survey participants. Most respondents indicated the use of dual‐lumen access catheters, although a wide variety of commercially available catheter types were employed (Figure 3).

### 3.2 Practice of ECCO_2_R

**FIGURE 4:**
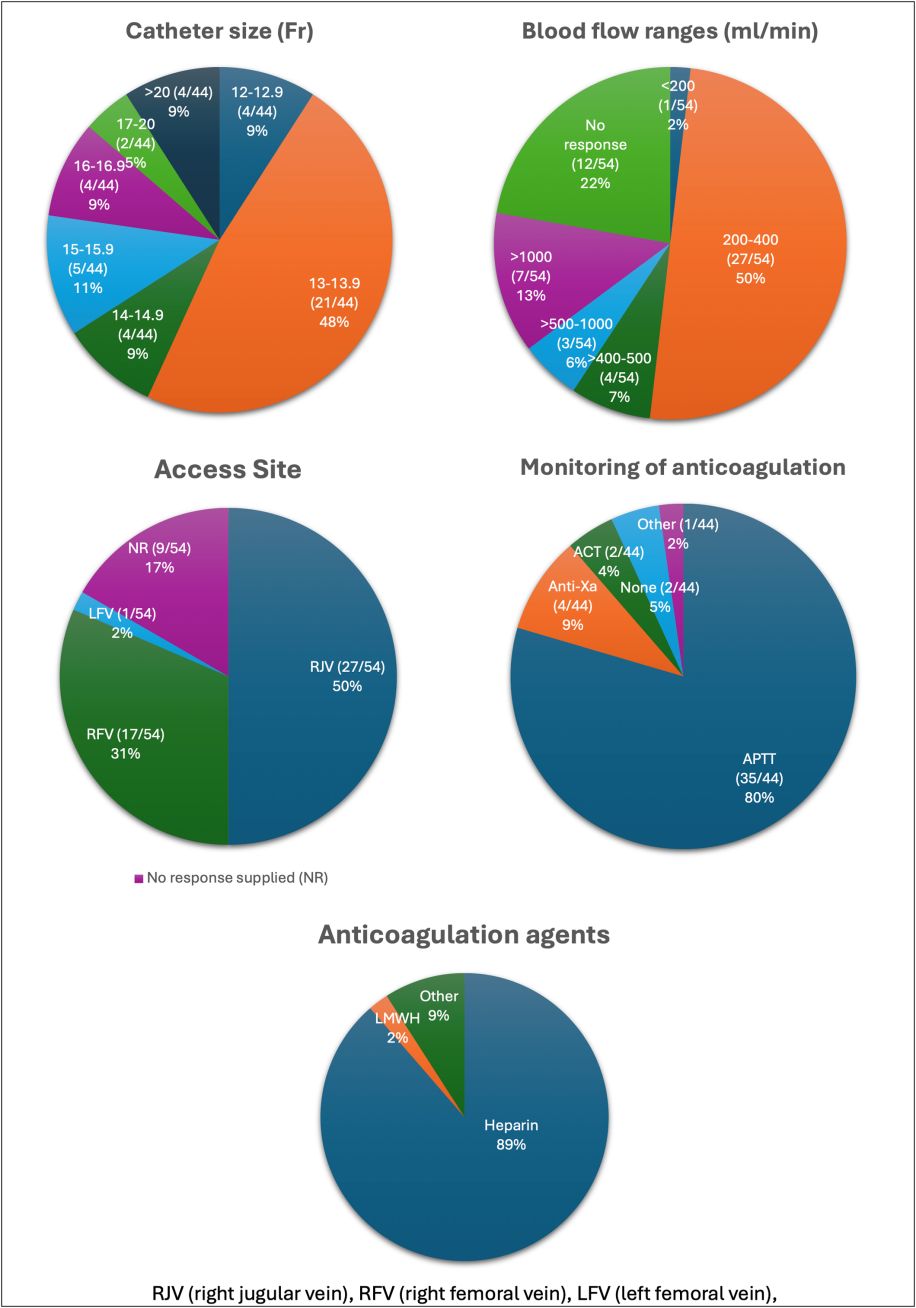
Practice of ECCO_2_R.

The responses based on current ECCO_2_R practice are shown in Figure 4. The preferred access site was the right jugular vein, followed by the right femoral vein. The catheter size and targeted blood flow ranged from 11F to > 20F and 200 to > 1000 mL/min, respectively. Heparin was used for anticoagulation by 89% of responders, and 2% reported using low molecular weight heparin. Monitoring of anticoagulation was mostly by APTT (80% of the responders) (Figure 4), and one‐third of respondents reported routine screening for haemolysis while ECCO_2_R was in use.

**FIGURE 5:**
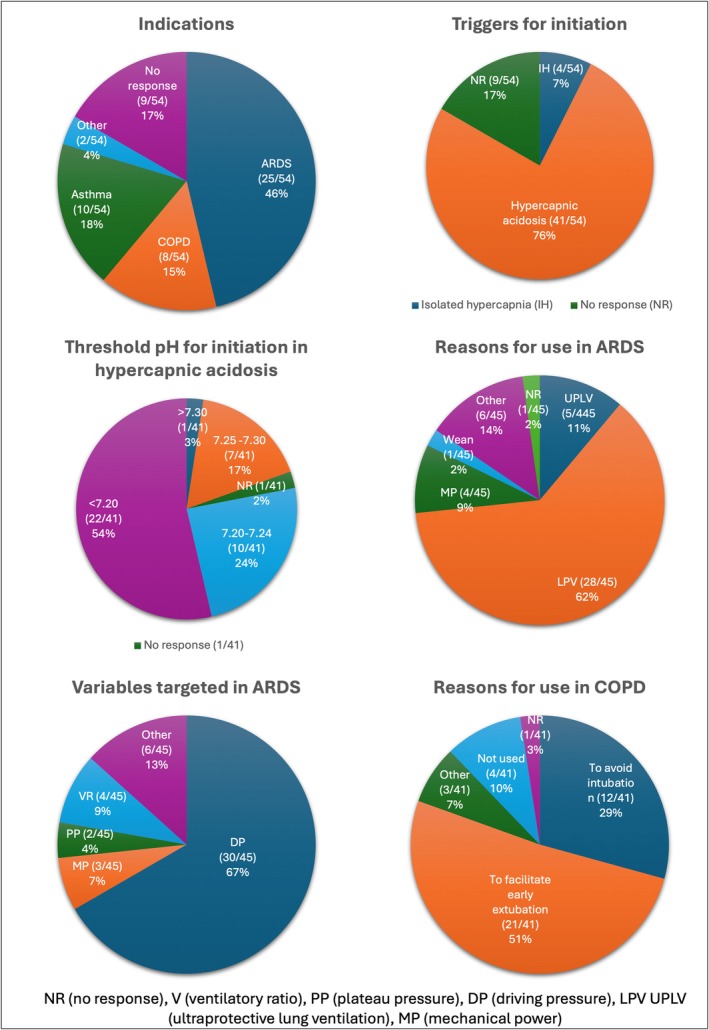
ECCO_2_R indications and targeted variables.

**FIGURE 6:**
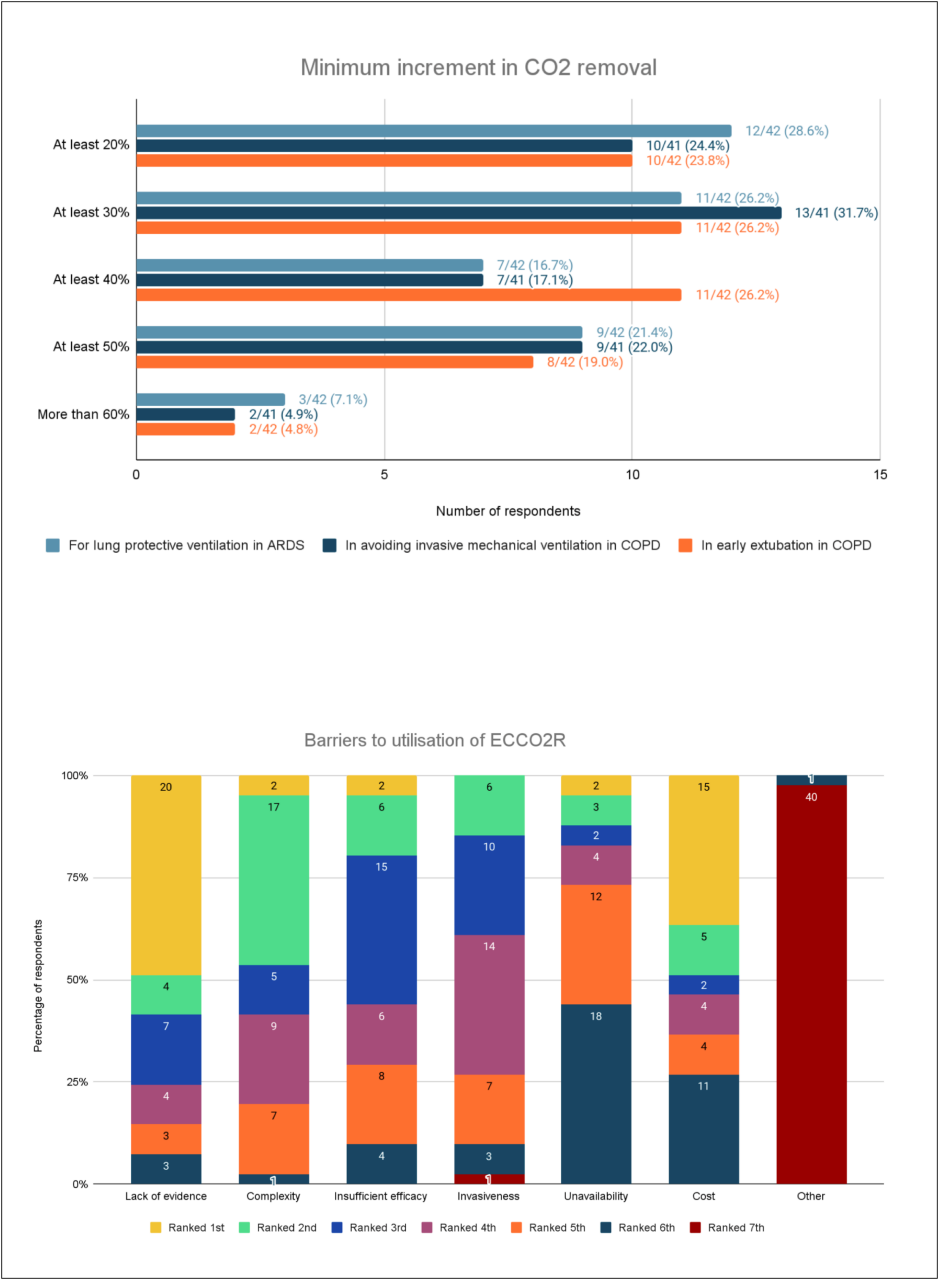
Increments in carbon dioxide removal effectiveness and barriers for the use of ECCO_2_R.

Figure 5 shows the reported indications and the targeted variables. ARDS management followed by asthma management was reported to be the common indicators—46% and 18%, respectively. Sixty‐seven percent of the respondents used ECCO_2_R to target a specific driving pressure in patients with ARDS. Triggers for initiation of ECCO_2_R were hypercapnic acidosis in 76% of the responders, and most reported a pH of < 7.2 as the threshold trigger for ECCO_2_R initiation. When respondents were questioned on the expected effectiveness of carbon dioxide removal for clinically useful ECCO_2_R, most responders reported that at least a 20%–30% increment in CO_2_ removal would be required. However, the responses were very varied (Figure 6). The reported barriers to wider utilisation of ECCO_2_R were lack of evidence, cost, complexity and lack of training (Figure 6).

### 3.3 ECCO_2_R Registry

**FIGURE 7:**
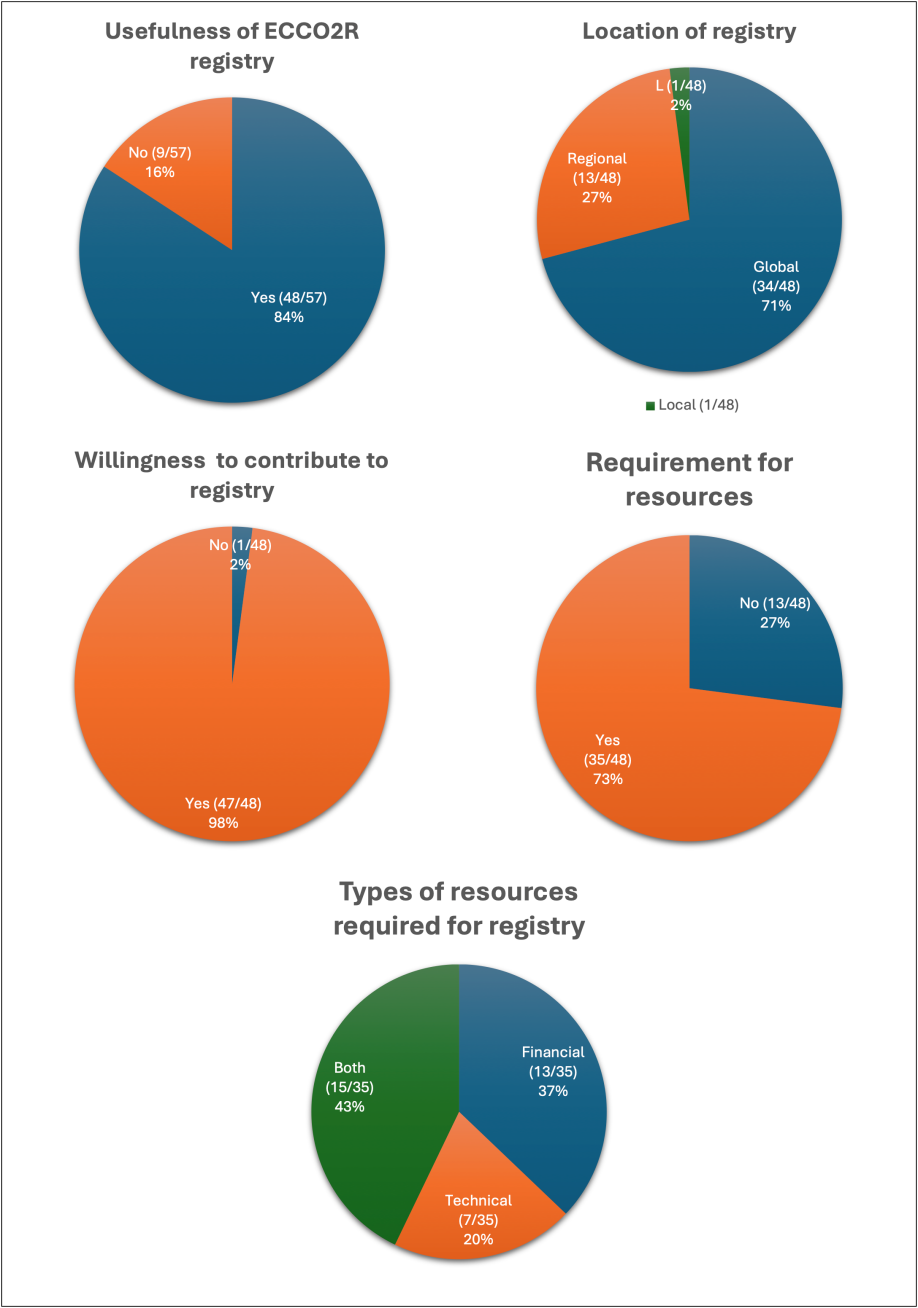
ECCO_2_R registry.

Eighty‐four percent of the respondents (48/57) thought an ECCO_2_R registry would be useful, and 98% (47/48) of the respondents reported that they would be willing to contribute to a dedicated ECCO_2_R registry. Nearly three‐quarters of the respondents (73%; 35/48) indicated they would need additional financial and technical resources to contribute to an ECCO_2_R registry (Figure 7).

## 4 Discussion

### 4.1 Key Findings

This survey included 70 responders from Europe, Asia, the Americas and Oceania. It demonstrated that the use of ECCO_2_R has significant variability in terms of the devices, vascular access catheters and the targeted blood flows. The most common use of ECCO_2_R was to support ventilation goals in ARDS management, followed by asthma management. Hypercapnic acidosis with a pH of less than 7.2 was the preferred trigger for the initiation of ECCO_2_R, and the most common reported barriers to use were lack of evidence, cost and complexity of the device. Eighty‐four percent of the respondents believe a registry for ECCO_2_R patients would be valuable and were willing to contribute to the registry with financial and technical support.

### 4.2 Relationship to Previous Findings

A survey on ECCO_2_R practices was conducted exclusively across French intensive care units [14] in 2015. The devices that were available at the time of the study were arteriovenous iLA (Novalung GmbH, Heilbronn, Germany) for 63% of the ECCO_2_R procedures and the Hemolung (Alung Technologies, Pittsburgh, PA) for 37%. The most typical indication of ECCO_2_R in their survey was for ARDS patients, which is similar to our survey findings. Our multinational survey, conducted almost a decade later, found many more devices in use and revealed that most users are employing veno‐venous ECCO_2_R; no participants reported arteriovenous ECCO_2_R in their practice.

The European ECCO_2_R consensus identifies driving pressure (≥ 14‐cm H_2_O), followed by plateau pressure (Pplat ≥ 25‐cm H_2_O), as the most important criteria for initiating ECCO_2_R in patients with ARDS, with the aim of improving case selection in future clinical trials of ECCO_2_R [15, 16].

Most participants reported using ECCO_2_R to target a specific driving pressure in the management of ARDS, consistent with consensus recommendations [16, 17], although this application has not yet been evaluated in randomised controlled trials.

Our survey found that the most common reason for not currently using ECCO_2_R was the lack of supporting evidence, unavailability of the device, followed by the cost and complications. These findings are unsurprising, as randomised controlled trials investigating the use of ECCO_2_R in the management of ARDS have demonstrated no mortality benefit and have reported increased morbidity, including fewer ventilator‐free days when ECCO_2_R was used [8]. Based on this data, the current European guidelines do not recommend the use of ECCO_2_R in ARDS patients outside randomised controlled trials [18].

More recently, a randomised controlled trial evaluating ECCO_2_R for the prevention of intubation or to facilitate early extubation in patients with COPD failed to demonstrate a benefit in facilitating early extubation. Moreover, the study showed harm when ECCO_2_R was used to prevent intubation, with an associated increase in all‐cause mortality [19]. In both randomised controlled trials of ARDS and COPD patients, more complications were noted in the patients on the ECCO_2_R arm.

In our survey, the targeted blood flow ranges varied from < 200 to > 1000 mL/min. The post hoc analyses of the SUPERNOVA trial [17] and the REST trial [20] suggest that higher dead space ventilation and ventilator ratios are key factors where ECCO_2_R use may potentially improve clinical outcomes [20]. It is uncertain if flows of 200 mL/min or a 20%–30% increment in CO_2_ removal would provide sufficient clearance of carbon dioxide to support lung protective or ultra‐protective ventilation to meaningfully improve the clinical outcomes [21]. Further research is required to establish the minimal CO_2_ removal when ECCO_2_R devices are used in patients with ARDS.

Registries of ECLS devices, such as ESLO, have helped define the use of ECMO by providing education, guidelines, quality assurance and high‐quality original research, which have helped to improve patient outcomes. Similar registries focusing on ECCO_2_R are likely to be important to aid better understanding and implementation of this therapy as well as informing the conduct of future randomised controlled trials [12, 13].

### 4.3 Implications of Study Findings

The findings of this survey, especially the evidence of practice variation such as blood flows and the type of devices, highlight a wide range of heterogeneity in the clinical application of ECCO_2_R. These practice variabilities represent a lack of consensus in how and when this technology should be used. Marked differences in clinical practice are typically associated with inconsistent outcomes and may limit the generalisability of findings from recent randomised controlled trials.

Anticoagulation management is a critical consideration in patients supported with extracorporeal devices. Recent randomised controlled trials have identified serious complications, including intracranial bleeding and major haemorrhage at other sites, such as haemothorax [8]. Collectively, these data emphasise the need to more clearly define the drivers of bleeding and to develop strategies to reduce this risk. Safer implementation of ECCO_2_R requires the establishment of quality clinical care guidelines and safety programmes, paralleling those available for renal replacement therapy and ECMO.

### 4.4 Significance

The concept of ECCO_2_R has been in clinical practice for over four decades with its first reported use in 1979. However, the technology to deliver ECCO_2_R has evolved significantly over the last decade along with variable practices. The potential benefit of ECCO_2_R depends on the balance between the safety and efficacy. While efficacy in terms of reduction in PaCO_2_ and mechanical ventilation was demonstrated in many studies [2, 22], the effectiveness in reducing mortality has not been demonstrated in recent randomised controlled trials [8, 19, 23]. The reported complication profile with ECCO_2_R is also highly variable [24], with some studies reporting significant (intracranial haemorrhage, ischaemic strokes, significant limb ischaemia or infections) adverse effects [8, 19, 25, 26], while other studies suggest no significant adverse effects related to ECCO_2_R [2, 5, 22, 27, 28]. This variability in safety and efficacy relates to the patient groups (including ARDS, COPD, asthma and other diagnostic categories), where ECCO_2_R is used as well as the device used for ECCO_2_R. Devices that used arteriovenous configuration had significant limb ischaemia as a complication [26], and devices with an integrated centrifugal pump showed a higher risk of haemolysis as well as intracranial events [8, 19]. Importantly, the centre experience in device application may also contribute to the complication profile, where experienced centres may report a lower complication profile [23] as compared to centres with limited experience in using ECCO_2_R devices [8]. Furthermore, the data on the cost effectiveness of ECCO_2_R are very limited. The cost utility analysis on the use of ECCO_2_R in the use of ARDS patients was associated with significantly higher costs, but no benefit in health‐related quality of life [10].

This multinational survey contributes to ongoing clinical discussion regarding the safety, efficacy and cost implications of ECCO_2_R. It highlights the need for greater consistency in practice to optimise clinical outcomes and improve resource utilisation. The lack of high‐quality randomised controlled trials to clearly identify patients most likely to benefit from ECCO_2_R, alongside existing evidence demonstrating no mortality benefit and the high cost of devices, remain key barriers to wider adoption.

## 5 Strengths and Limitations

### 5.1 Strengths

This is the first survey to evaluate current ECCO_2_R practices across the world following the recently published randomised controlled ECCO_2_R trials [10, 11, 23]. It covered current ECCO_2_R practices as well as clinician perceptions and their opinion about initiating a dedicated ECCO_2_R registry. Those who have published clinical research articles recently or have recent experience with low flow ECCO_2_R completed the surveys. The responses are therefore contemporary, informed and reflect both clinical and academic perception and practice of ECCO_2_R. The multinational scope of the survey enhances generalisability, making the findings applicable across various healthcare settings.

### 5.2 Limitations

The survey had a response rate of 50%. While this is considered a reasonable response rate [29], it does not eliminate bias or nonresponse errors. Mixed method administration of surveys including telephone, mail and web is known to improve the response rates, but the contact details available to us were largely email. This could have affected our survey response rate, although most clinicians use email as the primary mode of written communication. Our means of identifying participants could have introduced bias, since we approached known centres or clinicians currently practicing ECCO_2_R and identified these through published research in PubMed, through contact with experts and through company contacts of ECCO_2_R manufacturers. However, clinicians with sufficient experience on ECCO_2_R use can only answer the questions on the practice of ECCO_2_R, justifying the selection of the participants. This survey may have likely missed some clinicians who are currently practicing ECCO_2_R and has been skewed towards academic centres or programmes driven by key opinion leaders. Our survey did not capture whether centres no longer using ECCO_2_R now offer ECMO or no alternative extracorporeal support, and the potential for nonresponse bias limits interpretation of attitudes toward ECCO_2_R utility. The absence of structured questions addressing clinician attitudes and comparative perceptions of ECCO_2_R versus ECMO limits insight into decision‐making around ECCO_2_R adoption or abandonment and highlights an important area for future research.

## 6 Conclusions

There is substantial variation in ECCO_2_R practices and perceptions. More than 80% of the respondents believed a dedicated ECCO_2_R registry is needed. Establishing consistency of ECCO_2_R practices may optimise clinical outcomes and resource utilisation.

## Author Contributions

**Ravindranath Tiruvoipati:** conceptualization, methodology, analysis, original draft writing, project administration, review and editing. **Andrew Wang:** data curation, analysis, review and editing. **Matthew E. Cove:** draft review, revision and editing. **Son Do:** draft review, revision and editing. **QuangDai Huynh:** draft review, revision and editing. **Simon Sin Wai Ching:** draft review, revision and editing. All authors read and approved the final manuscript.

## Funding

The authors have nothing to report.

## Ethics Statement

Ethics approval was obtained from the Human Research and Ethics Committee of Peninsula Health (Project number: LNR/108005/PH‐2024). The need for informed consent was waived because of the observational nature of the study, and consent was implied by completion of the survey.

## Conflicts of Interest

R.T. was an invited speaker at several Baxter/Vantive‐sponsored meetings and has received a speaking fee. He also has an investigator initiated grant provided by Baxter Healthcare Pty Ltd for an ongoing research study.

A.W., M.E.C., S.D. and Q.H. declare no competing interest.

S.S.W.C. was an invited speaker at Baxter‐sponsored symposium and has received a speaking fee.

## Supporting information


**Appendix S1:** Invitation letter.

**Appendix S2:** Questions for survey.

## Data Availability

All data generated or analysed during this study are included in this published article.
