# Supplementary material for: Practice and Perceptions on Extracorporeal Carbon Dioxide Removal in the Current Era: A Multinational Survey
Source: Clin Respir J. 2026 Jun 16;20(6):e70203. doi: 10.1111/crj.70203 (PMC13270773; doi:10.1111/crj.70203)
Supplement: Supplementary file 1 — Appendix S1: Invitation letter. [file CRJ-20-e70203-s001.docx]

**Appendix 1; Invitation letter**

Dear [recipient],

You are invited to participate in a research survey conducted by the department of Intensive Care Medicine at Peninsula Health to obtain insights into the current utilisation of extracorporeal carbon dioxide removal (ECCO_2_R).

It consists of **about 20 short** **questions** and should take **approximately 10 minutes** to complete.

You could move away from a question that does not apply to your practice.

We would like you to participate in this survey. By completing the survey you:

- Consent to take part in the survey.
- Consent to the use of your survey data as described.
- Your participation in the survey is anonymous and voluntary.

You can access the survey by clicking the following link: [link]

In doing so, we thank you for your time and valuable contribution. The presentation of the results will be in aggregates and individual person, or centre will not be identified when reporting the results of this survey. The survey results is likely be published in a peer-review medical journal and a copy of the collated results can be provided to you on request.

Participation in this survey is voluntary, if you do not wish to take part you do not have to

This ethical aspects of this survey was reviewed and approved by Peninsula Health Human Research Ethics Committee (HREC Reference number: LNR/108005/PH-2024; SSA Reference number: LNRSSA/108005/PH-2024).

If you have any questions regarding this survey you may contact: Prof Ravindranath Tiruvoipati
(Telephone: +61 3 9784 7777; Email: travindranath@hotmail.com).

If you have any complaints about any aspect of this survey, the way it is being conducted or any questions about being a research participant in general you may contact: Manager, Office for Research Peninsula Health (Telephone: +61 3 9784 2679; Email: researchethics@phcn.vic.gov.au).

Kind regards,

ECCO_2_R survey team
