# Supplementary material for: Practice and Perceptions on Extracorporeal Carbon Dioxide Removal in the Current Era: A Multinational Survey
Source: Clin Respir J. 2026 Jun 16;20(6):e70203. doi: 10.1111/crj.70203 (PMC13270773; doi:10.1111/crj.70203)
Supplement: Supplementary file 2 — Appendix S2: Questions for survey. [file CRJ-20-e70203-s002.docx]

**Appendix 2: Questions for survey**

## Demographic data

1. Your location: Country: Institution/hospital:
2. Current Use:
3. Are you a current user of ECCO_2_R:

- Yes
- No (**please proceed to question 3**)

1. Years of experience of ECCO_2_R

- 0-2
- >2-5
- >5

1. *Device that you are currently using:*

- iLA/Nova Lung
- Hemolung
- PrismaLung+ (standalone ECCO_2_R)
- PrismaLung+ (RRT+ ECCO_2_R)
- multiECCO_2_R
- Other (Specify):

1. *Approximate number of ECCO_2_R cases per year in your unit*

- 0-10
- >10-20
- >20

1. *Past use:*
2. *Have you used ECCO_2_R in the past? (Answer only if not a current user):*

- *Yes*
- *No* **(please proceed to question 4)**
- *N/A* **(please proceed to question 4)**

1. *Device used in the past:*

- iLA/Nova Lung
- Hemolung
- PrismaLung+ (standalone ECCO_2_R)
- PrismaLung+ (RRT+ ECCO_2_R
- Other (Specify):

1. Years of experience in the past

- 0-2
- >2-5
- >5

1. *What is your reason for not using ECCO_2_R currently (select all that applies to you)?*

- *Lack of device available*
- *Unsure if the evidence support use of ECCO_2_R*
- *Costs*
- *Complications*
- *Others (Please specify)* -----

1. *Use of ECMO*
2. *Do you currently use ECMO in your unit?*

- Yes
- No (If no please proceed to question 5).

1. Do you use low flow ECMO specifically for ECCO_2_R?

- Yes
- No

1. *Years of experience in ECMO use*:

- 0-2 years
- >2-5 years
- >5 years

1. *Approximate number of ECCO_2_R using low flow ECMO cases per year in your unit:*

- 0-10
- 11-20
- >21

1. What flow rates do you target for low flow ECCO_2_R?

Free text -----

## Practice of ECCO_2_R (Answer only if you use a low flow ECCO_2_R DEVICE. NOT if ECCO_2_R is performed with low flow ECMO).

1. *Vascular access for ECCO2R*

*a. Preferred access site for ECCO_2_R (choose one)*

- Jugular
  - Right
  - Left
- Femoral
  - Right
  - Left
- b. Catheter size (Free text) -----
- c. Catheter type (Free text) ------
- d. Blood flow range on your ECCO_2_R (Free text) ------

1. *What is the most common indication for ECCO_2_R in your unit? (choose one)*

- ARDS management
- COPD management
- Asthma management

Other potential indication beyond, please specify (free text)

1. *Most common reason for utilisation in COPD management in your unit (choose one)*

- Avoid intubation
- Facilitate early extubation
- Other (please specify)

1. *Most common reason for utilisation in Asthma management in your unit (choose one)*

- Avoid intubation
- Facilitate early extubation
- Other (please specify)

1. *Most common reason for utilisation in ARDS (Choose one)*

- To facilitate ultra-protective lung ventilation (< 4 ml/kg tidal volume)
- Where lung protective ventilation (TV 6 ml/kg or driving pressure ≤13 cmH_2_O or plateau pressure <25 cmH_2_O) could not be maintained due to hypercapnic acidosis
- To reduce mechanical power
- To facilitate early extubation
- To facilitate early weaning off ECMO
- Other (please specify)
- *Would you specifically target any of the following variables in ARDS Patients on ECCO_2_R?*
- Yes
- No (if No proceed to question 10)

*If yes which do you target ( select all)*

- Driving pressure
  - (Target range?) (Free text Optional) -----
- Mechanical power
  - (Target range?) (Free text Optional) -----
- Ventilatory ratio
  - Preferred ratio (Free text) -----
- Other (please specify) Free text -----

1. *Anticoagulation agent used:*

- Heparin
- Low molecular weight heparin
- Other -----

1. *Specific anticoagulation monitoring while using ECCO_2_R*

- APTT
- Anti-Xa
- Activated clotting time (ACT)
- Other (Specify) -----

1. *Do you routinely perform haemolytic screen while patients are on ECCO_2_R*

- Yes
- No

1. *What are your triggers for initiation of ECCO_2_R?*
2. Hypercapnia (Isolated) (select one response)

- I do not use ECCO_2_R for hypercapnia if pH is >7.30
- I do not use ECCO_2_R for hypercapnia if pH is >7.25
- 45-60 mmHg
- 61-80 mmHg
- >81 mmHg

1. Hypercapnic acidosis (select one response)

- pH 7.30 or above with hypercapnia
- pH 7.25 -7.30 with hypercapnia
- pH 7.20-7.24 with hypercapnia
- pH<7.20 with hypercapnia

1. *Efficiency for lung protective ventilation in ARDS:*

What would be the Minimum increment in CO2 removal with the use of ECCO_2_R to be effective for lung protective ventilation in ARDS? (Select one response)

- At least 20% of CO_2_ elimination
- At least 30% CO_2_ elimination
- At least 40% CO_2_ elimination
- At least 50% CO_2_ elimination
- At least 60% CO_2_ elimination
- More than 60% CO_2_ elimination

1. *Efficiency for avoiding invasive ventilation in COPD:*

What would be the Minimum increment in CO2 removal with the use of ECCO2R to be effective in avoiding invasive mechanical ventilation in COPD? (Select one response)

- At least 20% of CO_2_ elimination
- At least 30% CO_2_ elimination
- At least 40% CO_2_ elimination
- At least 50% CO_2_ elimination
- At least 60% CO_2_ elimination
- More than 60% CO_2_ elimination

1. What would be the Minimum increment in CO_2_ removal with the use of ECCO_2_R to be effective in *early extubation in COPD?* (select one response)

- At least 20% of CO_2_ elimination
- At least 30% CO_2_ elimination
- At least 40% CO_2_ elimination
- At least 50% CO_2_ elimination
- At least 60% CO_2_ elimination
- More than 60% CO_2_ elimination

1. *What would you think are the barriers of ECCO_2_R for wider use in your region/city?*

*(Number applicable answers in their order of importance: “1” being most important)*

- __ Lack of evidence
- __ Complexity/lack of training on the use of device
- __Insufficient support with removal of carbon dioxide due to lower flows and lower CO_2_ extraction
- __Invasiveness and the associated complications
- __Unavailability of device
- __Costs with using the device
- __Other (please specify) -----

ECCO2R registry

1. Would there be value in an ECCO2R registry (like the ELSO Registry)?

- Yes
- No **(please proceed to question 18.)**

1. If yes, should such a registry be (select one):

- Global
- Regional (countries within a region)
- National (at country level)
- Local

1. Would you be willing to contribute your ECCO_2_R data to such a registry?

- Yes
- No

1. Would you need any resources to collate data to be submitted to a registry?

- Yes
- No **(please proceed to question 18.)**

1. If yes, which resources would you think will be required?

- Financial
- Technical (education on data points/data dictionary)
- Both

1. Do you currently submit data (non-ECCO_2_R) from your ICU for quality assurance to any of the databases (Local/regional or national)?

- Yes
- No

1. Do you need a copy of the results of this survey

- Yes
- No

If yes provide your email to which the results will be sent:----------------------------------
